# Phenotyping dividing cells in mouse models of neurodegenerative basal ganglia diseases

**DOI:** 10.1186/1471-2202-14-111

**Published:** 2013-10-03

**Authors:** Arthur Smardencas, Kerelos Rizkalla, Hyun Ah Kim, Jim Massalas, Claire O’Leary, Michelle E Ehrlich, Günter Schütz, Andrew J Lawrence, John Drago

**Affiliations:** 1 Florey Institute of Neuroscience and Mental Health, University of Melbourne, Melbourne, Australia; 2 Current address: Department of Pharmacology, Monash University, Clayton, Victoria, Australia; 3 Molecular and Cellular Therapeutics and RCSI Research Institute, Royal College of Surgeons in Ireland, Dublin, Ireland; 4 Department of Neurology, Mount Sinai School of Medicine, New York, USA; 5 Deutsches Krebsforschungszentrum, Heidelberg, Germany

**Keywords:** Striatum, D1-dopamine receptor, Huntington disease, Gene targeting, Neurodegeneration, Neurogenesis

## Abstract

**Background:**

Mice generated by a Cre/LoxP transgenic paradigm were used to model neurodegenerative basal ganglia disease of which Huntington disease (HD) is the prototypical example. In HD, death occurs in striatal projection neurons as well as cortical neurons. Cortical and striatal neurons that express the D1 dopamine receptor (Drd1a) degenerate in HD. The contribution that death of specific neuronal cell populations makes to the HD disease phenotype and the response of the brain to loss of defined cell subtypes is largely unknown.

**Methods:**

Drd1a-expressing cells were targeted for cell death and three independent lines generated; a striatal-restricted line, a cortical-restricted line and a global line in which Drd1a cells were deleted from both the striatum and cortex. Two independent experimental approaches were used. In the first, the proliferative marker Ki-67 was used to identify proliferating cells in eighty-week-old mice belonging to a generic global line, a global in which Drd1a cells express green fluorescent protein (GFP-global) and in eighty-week-old mice of a cortical line. In the second experiment, the proliferative response of four-week-old mice belonging to GFP-global and striatal lines was assessed using the thymidine analogue BrdU. The phenotype of proliferating cells was ascertained by double staining for BrdU and Olig2 (an oligodendrocyte marker), Iba1 (a microglial cell marker), S100β (an astroglial cell marker), or NeuN (a neuronal cell marker).

**Results:**

In the first study, we found that Ki-67-expressing cells were restricted to the striatal side of the lateral ventricles. Control mice had a greater number of Ki-67+ cells than mutant mice. There was no overlap between Ki-67 and GFP staining in control or mutant mice, suggesting that cells did not undergo cell division once they acquired a Drd1a phenotype. In contrast, in the second study we found that BrdU+ cells were identified throughout the cortex, striatum and periventricular region of control and mutant mice. Mutant mice from the GFP-global line showed increased BrdU+ cells in the cortex, striatum and periventricular region relative to control. Striatal line mutant mice had an increased number of BrdU+ cells in the striatum and periventricular region, but not the cortex. The number of microglia, astrocytes, oligodendrocytes and neurons generated from dividing progenitors was increased relative to control mice in most brain regions in mutant mice from the GFP-global line. In contrast, striatal line mutant mice displayed an increase only in the number of dividing microglia in striatal and periventricular regions.

**Conclusions:**

Genetically programmed post-natal ablation of Drd1a-expressing neurons is associated with an extensive proliferative response involving multiple cell lineages. The nature of the tissue response has the potential not only to remove cellular debris but also to forge physiologically meaningful brain repair. Age related deficits in proliferation are seen in mutant lines. A blunted endogenous reparative response may underlie the cumulative deficits characteristic of age related neurodegeneration.

## Background

Huntington disease (HD) is characterized by progressive degeneration and loss of neurons in the brain, particularly the dopamine-responsive medium spiny neurons of the striatum [1]. HD is an autosomal dominant condition caused by expansion of the polyglutamine repeat sequence in the protein Huntingtin. Clinically, HD patients may present with motor disorders, cognitive deficits, psychosis and anxiety disorder [2]. Mortality usually occurs within 15–20 years of onset of symptoms.

The D1 dopamine receptor (Drd1a)-expressing cell deletion mouse lines used in this study were generated by the Cre/LoxP transgenic method and include the global (i.e. striatal and cortical) (Drd1a/Tox × Cam Kinase IIa/Cre [3]), the striatal restricted (Drd1a/Tox × DARPP-32/Cre [4]), and cortical restricted (Drd1a/Tox × Emx-1/Cre) lines. The motor and neurochemical phenotype of individual lines depends on the molecular paradigm and target site from which Drd1a-expressing cells have been lost [3]. In this study, Cre recombinase-expressing transgenic mice are crossed with transgenic mice containing the attenuated diphtheria toxin (tox-176) gene “knocked into” the Drd1a gene locus downstream of a floxed NEOSTOP cassette [5]. Cell death follows Cre expression and Drd1a promoter driven tox-176 production at 1–2 weeks in the global line [3,6] and 5 weeks in the striatal line [4]. The Emx-1 promoter drives Cre expression during embryogenesis [7] and is selective for telencephalic pyramidal cells [8].

The anatomical changes that occur in the mammalian brain following sterile non-traumatic neuronal cell death are not completely understood. There is evidence from our laboratory that the density of D2 dopamine receptor-expressing striatal neurons increases following the loss of Drd1a-expressing neurons [3,5]. The response of other cell types in the brain such as oligodendrocytes, microglia and astrocytes remains to be defined.

The broad aim of the study was to characterize the proliferative response following targeted tox-176 mediated Drd1a cell injury. Two independent approaches were used; one of these approaches exploits the fact that proliferating cells express the protein Ki-67 in their nuclei. Another approach involved administration of the thymidine analogue 5’-bromo-2’-deoxyuridine (BrdU) as a marker for DNA synthesis. BrdU was used to assess proliferation in 4-week-old mice of the striatal and green fluorescent protein (GFP)-containing global [Drd1a-GFP/Tox × Cam Kinase IIa/Cre] lines (called GFP-global). In these mice, double labelling of BrdU with either NeuN (for neurons), Olig2 (to identify mature oligodendrocytes and oligodendrocyte progenitor cells), Iba1 (for microglia) or S100β (for astrocytes) was used to determine the quantity, distribution and phenotype of dividing cells in the brain. Eighty-week-old mice of the global (both GFP-expressing and non GFP-expressing) and cortical lines were assessed to determine the quantity and distribution of dividing cells. Double labelling with Ki-67 and GFP was used to determine if dividing cells were Drd1a-expressing.

## Results

### BrdU analysis

#### Cell distribution

##### Controls

**Figure 1:**
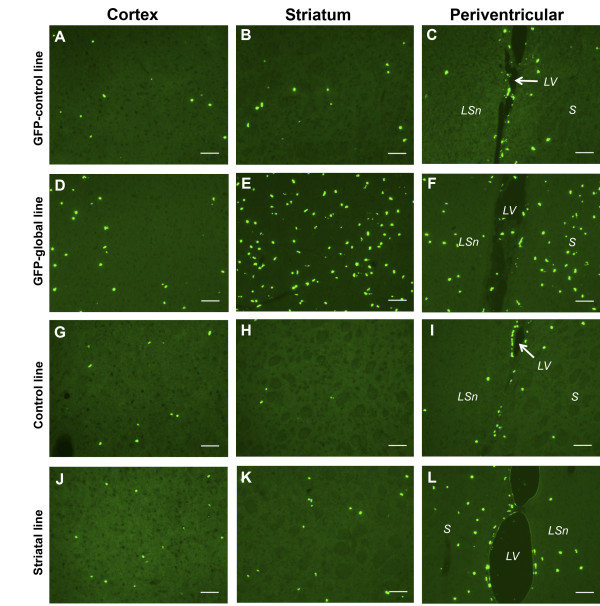
**Fluorescence microscopy of dividing cells in control and mutant mice.** Fluorescence photomicrographs showing BrdU+ (green) cells in the motor cortex **(A)**, striatum **(B)** and periventricular region **(C)** of a control mouse brain (GFP-control) of the GFP-global line. **(D-F)** Fluorescence micrographs showing BrdU+ (green) cells in the motor cortex **(D)**, striatum **(E)** and periventricular region **(F)** of a mutant mouse brain from the GFP-global line. **(G-I)** Motor cortex **(G)**, striatum **(H)** and periventricular region **(I)** of a control mouse brain of the striatal line. **(J-L)** Fluorescence micrographs showing BrdU+ (green) cells in the motor cortex **(J)**, striatum **(K)** and periventricular region **(L)** of a mutant mouse brain from the striatal line. *S* denotes striatum, *LSn* denotes lateral septal nucleus and *LV* denotes lateral ventricle. Scale bar represents 70 μm.

Cells positive for BrdU were identified in control mice for the two lines examined (GFP-global and striatal). The BrdU+ cells were distributed throughout the motor cortex, striatum and periventricular region. Figure 1 (A-C) and Figure 1 (G-I) are representative photomicrographs of coronal sections showing BrdU+ cells throughout three brain regions in control mice for GFP-global (GFP-control) and striatal lines respectively.

Cells that were positive for Iba1, Olig2, S100β and NeuN were also present throughout the cortex, striatum and periventricular region of control mice for both lines (see below). Double staining showed that Iba1+/BrdU+, Olig2+/BrdU+, S100β+/BrdU+ and NeuN+/BrdU+ cells were present throughout all three regions.

##### Mutant mice

BrdU+ cells were also distributed throughout the motor cortex, striatum and periventricular region of mutant mice belonging to the GFP-global and striatal lines. Figure 1 (D-F) and Figure 1 (J-L) are representative photomicrographs of coronal sections showing BrdU+ cells throughout the three regions of the mutant brain in the GFP-global and striatal lines respectively. Cells that were positive for Iba1, Olig2, S100β and NeuN were also present throughout the cortex, striatum and periventricular region of both mutant lines (see below), while double staining showed that Iba1+/BrdU+, Olig2+/BrdU+, S100β+/BrdU+ and NeuN+/BrdU+ cells were also present throughout these regions.

#### Cell quantification

##### GFP-global line; BrdU+ cells

**Figure 2:**
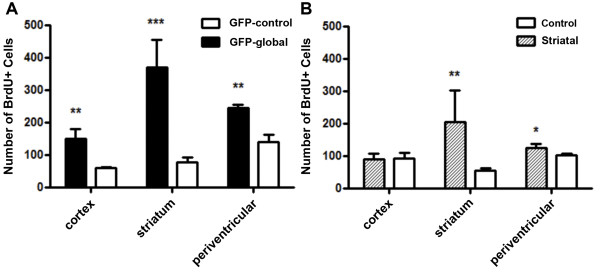
**Regional quantification of dividing cells in GFP-control and GFP-global mutant mice.** Bar graphs showing the number of BrdU+ cells in each brain region of control (GFP-control) and mutant mice from the GFP-global **(A)**, and control and striatal lines **(B)**. Each bar represents the mean (± SEM) of the cumulative number of BrdU+ cells per mutant (*n*=4) or control (*n*=4) as determined from five coronal sections per mouse obtained between 1.18 mm to −0.46 mm from bregma. Asterisks refer to the results of the 2-way ANOVA using the original cell counts. There was a main effect of genotype in the form of a greater number of BrdU+ cells in the cortex (**P<0.01), striatum (***P≤0.0001) and periventricular region (**P<0.01) of the GFP-global relative to GFP-control lines. There was a main effect of genotype in the form of a greater number of BrdU+ cells in the striatum (*P<0.05) and periventricular region (*P<0.05) of striatal line relative to control mice.

Regional quantification of the number of BrdU+ cells was undertaken in the GFP-global line and GFP-control mice. A two-way ANOVA demonstrated no significant genotype-by-bregma level interaction in the cortex, striatum or periventricular region of GFP-control (*n*=4) and GFP-global (*n*=4) mice. There was a main effect of genotype, on the BrdU+ cell count in the cortex (*P*<0.01), striatum (*P*≤0.0001) and periventricular region (*P*<0.01); the BrdU+ cell count was increased in mutant relative to control mice in each of these regions. Figure 2A shows the mean (± SEM) of the cumulative number of BrdU+ cells for specific regions - where cumulative number refers to the sum of the BrdU+ cells from the five bregma levels (1.18, 0.62, 0.26, 0.02 and −0.46) quantified for each animal.

##### GFP-global line; NeuN+/BrdU+ cells

**Figure 3:**
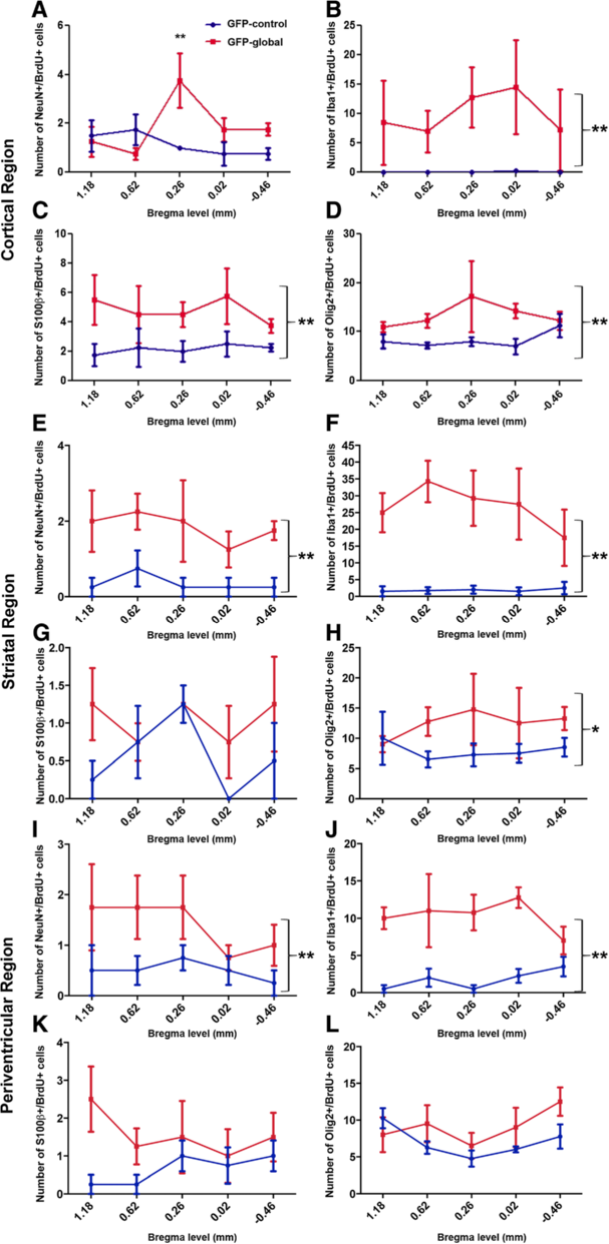
**Regional quantification of neuronal, microglial, astroglial and oligodendroglial lineage cells derived from dividing progenitors in GFP-control and GFP-global mutant mice.** Graphs showing the number of double-positive NeuN+/BrdU+, Iba1+/BrdU+, S100β+/BrdU+ and Olig2+/BrdU+ cells at specific sites relative to bregma in the M1 motor cortex **(A-D)**, striatum **(E-H)** and periventricular **(I-L)** regions of the brain of mice belonging to the GFP-global line and GFP-control mice injected for 2 weeks with BrdU+ at 4 weeks of age and killed at age 8 weeks of age for analysis. Each point represents the mean ± SEM as determined for each bregma level in mutant (n=4) and control (n=4) mice. There was a greater number of NeuN+/BrdU+ cells in GFP-global mutant relative to control mice measured at 0.26mm from bregma (**P<0.01) as determined by a Bonferroni post-hoc test. There was also a significant increase in the number of NeuN+/BrdU+ cells in the striatum (*P*< 0.01) and periventricular regions (*P*< 0.01), Iba1+/BrdU+ cells in all three regions (all *P*< 0.01), S100β+/BrdU+ cells in the cortex (*P*< 0.01) and periventricular regions (*P*< 0.01) and Olig2+/BrdU+ cells in the cortex (*P*< 0.01) and striatum (*P*< 0.05) in GFP-global mutant relative to control mice.

Regional quantification of the number of NeuN+/BrdU+ cells was undertaken in the GFP-global line and GFP-control mice. A two-way ANOVA demonstrated a significant genotype-by-bregma level interaction (*P*<0.05) in the cortex of GFP-control (*n*=4) and GFP-global (*n*=4) mice (Figure 3A). A Bonferroni post-hoc test revealed a significant increase in the NeuN+/BrdU+ cell count in mutant relative to control mice at bregma level 0.26 mm (*P*<0.01). In both the striatum (Figure 3E), and the periventricular (Figure 3I) region of these mice there was a main effect of genotype (both *P*<0.01) but no genotype-by-bregma level interaction, indicating that the two genotypes always differed irrespective of bregma level. Table 1 shows the mean (± SEM) of the percentage of BrdU+ cells that were NeuN+.

**Table 1:** The mean (± SEM) of the percentage of BrdU+ cells that are Iba1+, S100β+, Olig2+ and NeuN+ per animal in the defined regions of the brain of GFP-global mutant (n=4) and GFP-control (n=4) mice

	***Motor Cortex***		***Striatum***		***Periventricular Region***	
	**Mutant**	**Control**	**Mutant**	**Control**	**Mutant**	**Control**
**Iba1+**	23.2 ± 7.9	0.4 ± 0.4	37.7 ± 5.6	12.0 ± 6.3	22.5 ± 2.3	6.8 ± 1.4
**S100β+**	18.5 ± 4.9	16.9 ± 4.2	1.8 ± 0.7	2.7 ± 0.5	2.7 ± 0.1	2.5 ± 0.9
**Olig2+**	49.8 ± 9.8	78.3 ± 1.1	17.3 ± 1.4	58.2 ± 11.3	21.4 ± 2.9	26.0 ± 3.9
**NeuN+**	7.8 ± 0.9	9.2 ± 2.1	2.2 ± 0.2	1.5 ± 0.5	2.9 ± 0.8	1.7 ± 0.7
Marker-negative*	0.7	0	41.0	25.6	50.5	63.0

* refers to the percentage of BrdU+ cells that did not label with any of the four antibodies. This was calculated as the difference between the sum of the percentages for the panel of four antibodies and 100. This calculation assumes that the selected lineage specific antibodies are indeed specific and BrdU positive cells in this study co-express only one lineage marker.

##### GFP-global line; Iba1+/BrdU+ cells

Regional quantification of the number of Iba1+/BrdU+ cells was undertaken in the GFP-global line and GFP-control mice. A two-way ANOVA demonstrated a main effect of genotype in the cortex, striatum and periventricular region between control (*n*=4) and mutant (*n*=4) mice (all *P*<0.01) but no genotype-by-bregma level interaction in any region (Figure 3B, E and J). Table 1 shows the mean (± SEM) of the percentage of BrdU+ cells that were Iba1+ per animal in all regions of the brain for both mutant and control mice.

##### GFP-global line; S100β+/ BrdU+ cells

**Figure 4:**
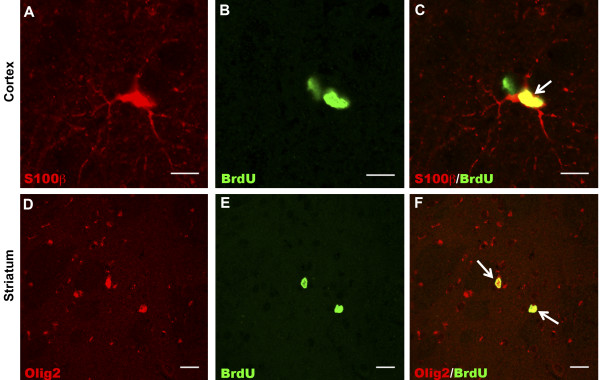
**Fluorescence microscopy of astroglial and oligodendroglial lineage cells derived from dividing cells.** Panel **A** is a confocal photomicrograph that shows an S100β+ (red) astroglial cell in the cortex of a GFP-global line mouse brain. Panel **B** is a confocal photomicrograph that shows BrdU+ (green) cells in the same field. Panel **C** represents the merging of panels **A** and **B** and indicates the cell (arrow) is a double positive S100β+/BrdU+ (yellow) astroglial cell, which has undergone cell division. Scale bar in panels **A**, **B** and **C** represents 15 μm. Panel **D** is a confocal photomicrograph showing Olig2+ (red) cells in the striatum of a mutant mouse brain from the GFP-global line. Panel **E** is a confocal photomicrograph that shows BrdU+ (green) cells in the same field. Panel **F** represents the merging of panels **D** and **E** and indicates that two cells (arrows) are Olig2+/BrdU+ (yellow) and are therefore oligodendroglial lineage cells, which have undergone cell division. Scale bar in panels **D**, **E** and **F** represents 12 μm.

Figure 4A-C depicts confocal photomicrographs showing S100β+ and BrdU+ cells and an S100β+/BrdU+ double positive cell in the cortex of a GFP-global mutant mouse brain. Regional quantification of the number of S100β+/BrdU+ cells was undertaken in the GFP-global line (*n*=4) and control mice (*n*=4). A two-way ANOVA demonstrated a main effect of genotype in the cortex (*P*<0.01) (Figure 3C) and periventricular region (*P*<0.05) (Figure 3K), but not in the striatum (Figure 3G). There was no significant genotype-by-bregma level interaction in any region. Table 1 shows the mean (± SEM) of the percentage of BrdU+ cells that was S100β+ per animal in all regions of the brain for both mutant and control mice.

##### GFP-global line; Olig2+/BrdU+ cells

Figure 4 depicts confocal photomicrographs of Olig2+, BrdU+ cells and Olig2+/BrdU+ double positive cells in the striatum of a mutant GFP-global brain. Regional quantification of the number of Olig2+/BrdU+ cells was undertaken in the GFP-global line (*n*=4) and GFP-control mice (*n*=4). A two-way ANOVA demonstrated a main effect of genotype in the cortex (*P*<0.01) (Figure 3D) and striatum (*P*<0.05) (Figure 3H) but not in the periventricular region (Figure 3L). There was no significant genotype-by-bregma level interaction in any region. Table 1 shows the mean (± SEM) of the percentage of BrdU+ cells that were Olig2+ per animal in all regions of the brain for both mutant and control mice.

##### Striatal line; BrdU+ cells

Regional quantification of the number of BrdU+ cells was undertaken in the striatal line (*n*=4) and littermate control mice (*n*=4). A two-way ANOVA demonstrated a main effect of genotype on the number of BrdU+ cells in the striatum (*P*<0.01) and periventricular region (*P*<0.05); the BrdU+ cell count was significantly increased in mutant relative to control mice in each region. There was no effect of genotype or bregma level on the BrdU+ cell count in the cortex. There was no significant genotype-by-bregma level interaction in any region. Figure 2B shows the mean (± SEM) of the cumulative number of BrdU+ cells per animal in each region of the brain for mutant and control mice.

##### Striatal line; NeuN+/BrdU+ cells

**Figure 5:**
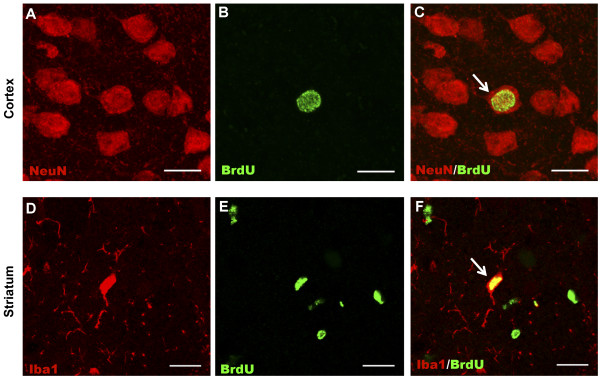
**Fluorescence microscopy of neuronal and microglial cells derived from dividing progenitors.** Panel **A** is a confocal photomicrograph that shows NeuN+ (red) cells in the cortex of a control mouse brain for the striatal line. Panel **B** is a confocal photomicrograph that shows a BrdU+ (green) cell in the same field. Panel **C** represents the merging of panels **A** and **B**, and indicates that the cell (arrow) is NeuN+/BrdU+ (yellow) and therefore represents a neuron derived from a dividing progenitor cell. Scale bar in panels **A**, **B** and **C** represents 12 μm. Panel **D** is a confocal photomicrograph that shows Iba1+ (red) cells in the striatum of a mutant mouse brain from the striatal line. Panel **E** is a confocal photomicrograph that shows BrdU+ (green) cells in the same field. Panel F represents the merging of panels **D** and **E** and indicates that one cell (arrow) is Iba1+/BrdU+ (yellow) and therefore represents a microglial cell derived from a cell that has undergone cell division. Scale bar in panels **D**, **E** and **F** represents 15 μm.

**Figure 6:**
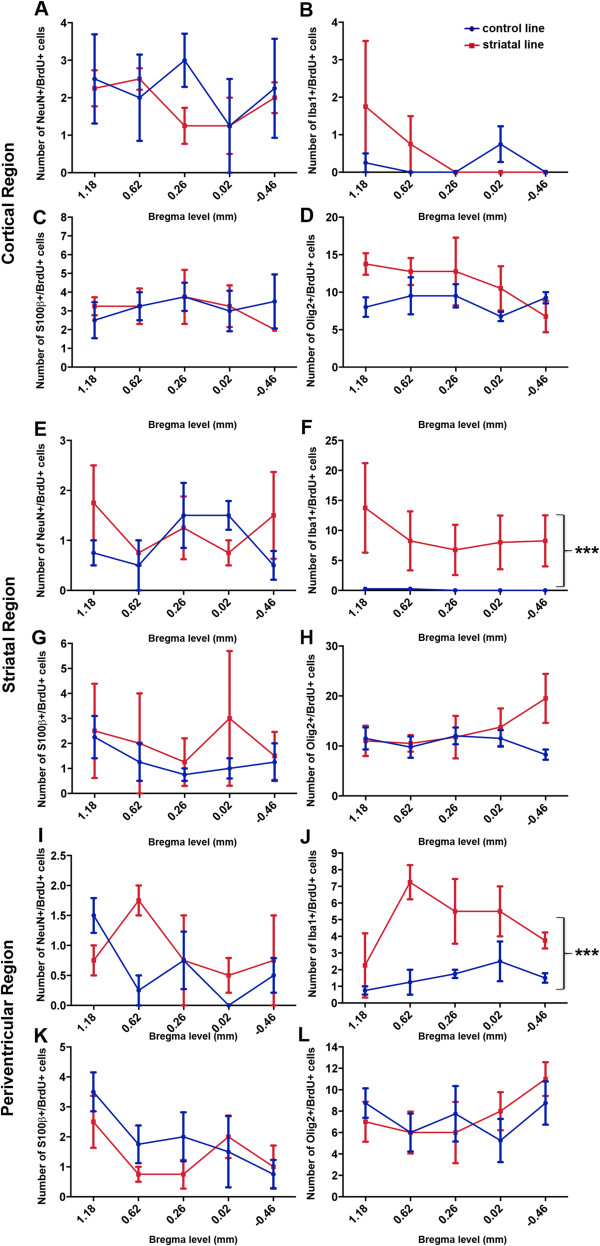
**Regional quantification of neuronal, microglial, astroglial, and oligodendroglial cells derived from dividing progenitors in control and striatal line mutant mice.** Graphs showing the number of double-positive NeuN+/BrdU+, Iba1+/BrdU+, S100β+/BrdU+ and Olig2+/BrdU+ cells at specific sites relative to bregma in the M1 motor cortex **(A-D)**, striatum **(E-H)** and periventricular **(I-L)** regions of the brain of mice belonging to the striatal line and control mice injected for 2 weeks with BrdU+ at 4 weeks of age and killed at age 8 weeks. There was a significant increase in the number of Iba1+/BrdU+ cells in the striatum and periventricular regions (both *P*< 0.001) in striatal mutant relative to control mice.

Figure 5 represents confocal photomicrographs showing NeuN+, BrdU+ and a NeuN/BrdU double positive cell in the cortex of a control brain. Regional quantification of the number of NeuN+/BrdU+ cells was undertaken in the striatal line and control mice. A two-way ANOVA demonstrated no significant genotype-by-bregma level interaction in the cortex (Figure 6A), striatum (Figure 6E) or periventricular (Figure 6I) region of control (*n*=4) and mutant (*n*=4) mice. Furthermore, there was no main effect of genotype in any region. Table 2 shows the mean (± SEM) of the percentage of BrdU+ cells that were NeuN+ per animal in all regions of the brain for mutant and control mice.

**Table 2:** **The mean (± SEM) of the percentage of BrdU+ cells that are also Iba1+, S100β+, Olig2+ and NeuN+ per animal in defined brain regions of the brain of striatal mutant (*n *=4) and control (*n *=4) mice**

	***Motor Cortex***		***Striatum***		***Periventricular Region***	
	**Mutant**	**Control**	**Mutant**	**Control**	**Mutant**	**Control**
**Iba1+**	1.8 ± 1.8	0.9 ± 0.7	19.8 ± 1. 9	0.8 ± 0.5	18.8 ± 2.4	8.5 ± 0.7
**S100β+**	22.1 ± 1.9	21.6 ± 1.9	3.7 ± 2.0	13.3 ± 2.5	5.9 ± 1.5	8.1 ± 1.3
**Olig2+**	74.8 ± 2.1	70.9 ± 3.2	46.1 ± 8.4	72.6 ± 4.8	32.4 ± 3.5	31.3 ± 5.8
**NeuN+**	14.7 ± 1.3	14.3 ± 6.1	3.2 ± 0.2	8.1 ± 1.9	3.5 ± 0.9	2.5 ± 0.5
Marker-negative*	0	0	27.2	5.2	39.4	49.6

* refers to the percentage of BrdU+ cells that did not label with any of the four antibodies. This was calculated as the difference between the sum of the percentages for the panel of four antibodies and 100. This calculation assumes that the selected lineage specific antibodies are indeed specific and BrdU positive cells in this study co-express only one lineage marker.

##### Striatal line; Iba1+/BrdU+ cells

Figure 5(D-F) represents confocal photomicrographs showing Iba1+, BrdU+ cells and an Iba1/BrdU double positive cell in the striatum of a striatal-line mutant brain. Regional quantification of the number of Iba1+/BrdU+ cells was undertaken in the striatal line (*n*=4) and control mice (*n*=4). A two-way ANOVA demonstrated a main effect of genotype in the striatum (Figure 6F) and periventricular region (Figure 6J) (both *P*<0.001), but no effect in the cortex (Figure 6B). There was no significant genotype-by-bregma level interaction in any region. Table 2 shows the mean (± SEM) of the percentage of BrdU+ cells that were Iba1+ per animal in all regions of the brain for both mutant and control mice.

##### Striatal line; S100β+/BrdU+ cells

Regional quantification of the number of S100β+/BrdU+ cells was undertaken in the striatal line and control mice. A two-way ANOVA demonstrated no significant genotype-by-bregma level interaction in the cortex, striatum or periventricular region of control (*n*=4) and mutant (*n*=4) mice. Furthermore, there was no main effect of genotype in any region. Table 2 shows the mean (± SEM) of the percentage of BrdU+ cells that were S100β+ per animal in all regions of the brain for mutant and control mice.

##### Striatal line; Olig2+/BrdU+ cells

Regional quantification of the number of Olig2+/BrdU+ cells was undertaken in the striatal line and control mice. A two-way ANOVA demonstrated no significant genotype-by-bregma level interaction in the cortex, striatum or periventricular region of control (*n*=4) and mutant (*n*=4) mice. There was no effect of genotype on the number of Olig2+/BrdU+ cells in any region (Figure 6D, H and L). Table 2 shows the mean (± SEM) of the percentage of BrdU+ cells that were Olig2+ per animal in all regions of the brain.

### Summary of the BrdU-co-labelling analyses

The young mutant GFP-global and striatal lines demonstrated a statistically significant increase in BrdU+ cell numbers relative to control mice. There was a difference between the two mutant lines in the regional distribution of dividing cells. The GFP-global line displayed an increase in the number of BrdU+ cells in the cortex, striatum and periventricular region. In contrast, up-regulated BrdU+ cell numbers were restricted to the striatum and periventricular region in the striatal ablation line. Significant increases in double-labelled cell numbers were observed in mutant relative to control mice; however, a difference was observed between the two mutant lines. GFP-global line mutant mice displayed increased NeuN+/BrdU+, Iba1+/BrdU+, S100β+/BrdU+ and Olig2+/BrdU+ cell numbers in at least two of the three regions of the brain, including the cortex whereas striatal line mutant mice only displayed an increase in the Iba1+/BrdU+ cell numbers with this increase being confined to the striatum and periventricular region.

### Ki-67 analysis

#### Cell distribution

**Figure 7:**
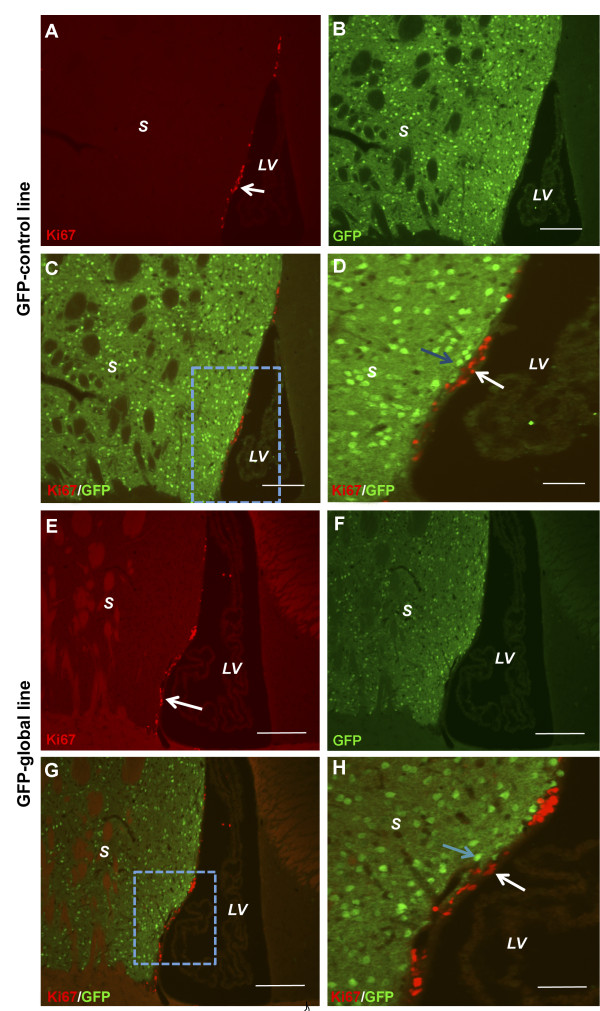
**Fluorescence photomicrographs showing Ki-67+ (red) and GFP+ (green) cells in GFP-control and GFP-global lines analysed at 80 weeks post-natal.** In GFP-control mice, **(A)** Ki-67+ cells (arrow) are located along the striatal side of the lateral ventricle and **(B)** GFP staining shows Drd1a-expressing cells and dense neuropil distributed throughout the striatum. There is no overlap between Ki-67+ (white arrow) and GFP+ (blue arrow) cells (panels **C** and **D**). Scale bar represents 150 μm in panels **A**, **B**, **C**, and 50 μm in panel **D**. In GFP-global line mice, Ki-67+ cells (arrow) are distributed along the striatal side of the lateral ventricle **(E)** and GFP staining shows Drd1a-expressing cells and neuropil distributed throughout the striatum **(F)**. As for GFP-control mice, no overlap of Ki-67+ (white arrow) and GFP+ (blue arrow) cells (**G** and **H**) was observed in GFP-global line mice. *S* denotes striatum and *LV* denotes lateral ventricle. Scale bar represents 150μm in panels **E**, **F**, **G**, and 50μm in panel **F**.

Immunohistochemical staining for Ki-67+ cells revealed an irregular distribution pattern throughout the periventricular region lining the lateral ventricles in littermate control mice. Ki-67+ cells were identified only on the striatal side of the ventricles. Figure 7 shows photomicrographs of a GFP-control brain. The Ki-67+ cells line the lateral ventricles (Figure 7A). The GFP+ cells were distributed throughout the entire striatum (Figure 7B). Double staining showed that there were no cells positive for both Ki-67 and GFP (Figures 7C and 7D). Ki-67+ cells were also distributed in an irregular pattern throughout the striatal side of the periventricular region lining the lateral ventricles in mutant global, GFP-global and cortical lines. Figure 7(E-H) depicts a photomicrograph of a mutant brain from a GFP-global line. The Ki-67+ cells line the lateral ventricles (Figure 7E). GFP+ cells were distributed throughout the entire striatum (Figure 7F). Double staining showed that there were no cells positive for both Ki-67 and GFP (Figures 7G and 7H).

#### *Cell quantification*; GFP-global line

**Figure 8:**
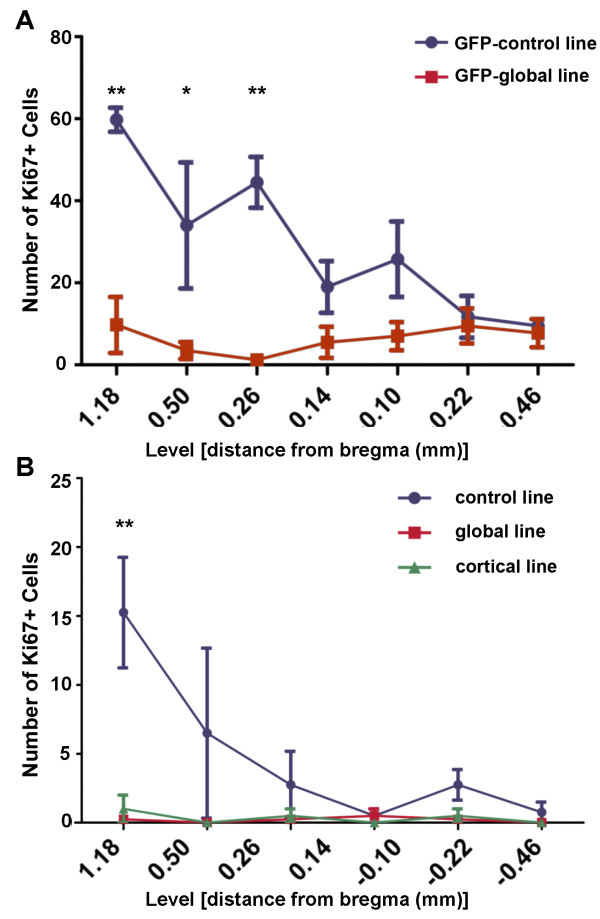
**Regional quantification of Ki67+ cells in GFP-control, GFP-global, global-mutant, cortical-mutant and control lines. (A)** The distribution of Ki-67+ cells relative to bregma on the striatal side of the periventricular region in the brain of GFP-global mutant and GFP-control mice analysed at 80 weeks post-natal. Each point represents the mean ± SEM at a specific distance from bregma in mutant (*n*=4) and control (*n*=4) mice. There were fewer Ki-67+ cells in mutant mice, the difference being significant at the three most cranially located points of 1.18 (**P<0.01), 0.50 (*P<0.05) and 0.26 (**P<0.01) mm from bregma as determined by a Bonferroni post-hoc test. **(B)** The distribution of Ki-67+ cells relative to bregma in the striatal side of the periventricular region of the brain of mutant mice belonging to the global and cortical lines and global line littermate age matched control mice. Each point represents the mean ± SEM as determined for each distance from bregma in mutant (*n*=4) and control (*n*=4) mice. In each line, there were fewer Ki-67+ cells in mutant relative to control mice with the difference being significant at 1.18 mm from bregma (**P<0.01) as determined by a Bonferroni post-hoc test.

A two-way ANOVA demonstrated a significant (*P*<0.01) genotype-by-bregma level interaction in the periventricular region of control (*n*=4) and mutant (*n*=4) mice. A Bonferroni post-hoc test confirmed a significant reduction in the Ki-67+ cell count in mutant relative to control mice at bregma levels 1.18 mm (*P*<0.01), 0.50 mm (*P*<0.05) and 0.26 mm (*P*<0.01). Figure 8A shows the mean (± SEM) of the Ki-67+ cell count at each bregma level for control and mutant mice.

#### *Cell quantification*; global line

A two-way ANOVA demonstrated a significant (*P*<0.05) genotype-by-bregma level interaction in the periventricular region of control (*n*=4) and mutant (*n*=4) mice. A Bonferroni post-hoc test showed that there was a significant reduction in the Ki-67+ cell count in mutant relative to control mice at bregma level of 1.18 mm (*P*<0.01). Figure 8B shows the mean (± SEM) of the Ki-67+ cell count at each bregma level for the control and mutant mice.

#### Cell quantification; cortical line

A two-way ANOVA demonstrated a significant (P<0.05) genotype-by-bregma level interaction in the periventricular region of control (*n*=4) and mutant (*n*=4) mice. A Bonferroni post-hoc test showed that there was a significant reduction in the Ki-67+ cell count in mutant relative to control mice at bregma level 1.18 mm (*P*<0.01). Figure 8B shows the mean (± SEM) of the Ki-67+ cell count at each bregma level for control and mutant mice.

## Discussion

We observed a marked increase of BrdU+ cell numbers in young mutant mice relative to control mice in the GFP-global and striatal lines. We saw an increase in the number of BrdU+ cells in brain regions, predicted on the basis of the Cre activator line used, to sustain maximal levels of tox-176-mediated death of Drd1a-expressing cells. These regions are the cortex and striatum (including the periventricular region) of GFP-global line mutant mice, and the striatum (including the periventricular region) of striatal line mutant mice. As BrdU is a marker of DNA synthesis [9], our study suggests that the brain produces new, dividing cells in an attempt to compensate for the regionally restricted tox-176-mediated loss of Drd1a-expressing cells. A limitation of BrdU as a proliferative marker is that it not only incorporates into nascent DNA during S phase of the mitotic cycle but it may also integrate into cellular DNA during DNA repair and in the context of apoptosis. Although both DNA repair and apoptosis represent theoretical possibilities, this would not explain the uniform nuclear staining, the number of BrdU+ doublets, and more importantly the large number of BrdU positive cells (~ 720 cells/5 sections-see Figure 2A and Figure 1D-F) seen in global ablation mutant mice. Furthermore, TUNEL staining undertaken in global ablation mutant mice [3] aged between 2 and 9 weeks identified very few TUNEL positive cells. In addition, TUNEL-positive cells were not seen in the cortex. A small number of TUNEL-positive cells (approximately five/section) were seen in the hippocampus and thalamus of a two-week old mutant, brain regions known to express the Drd1a-receptor. The degree of striatal volume loss is no different in the striatal-restricted line and the mechanism of cell death is the same (i.e. tox-176 mediated cell death) and so there is no reason to believe that apoptosis would be any more prominent in this line. Taken together, these data suggest that it is highly improbable that the large number of BrdU-positive cells seen in our mutant lines represent anything other than active cell turnover in response to the targeted attenuated diphtheria-toxin mediated cell death of Drd1a-expressing cells.

There was also a marked increase in the number of dividing microglia/macrophages in young mutant mice belonging to the GFP-global and striatal lines. This increase was confined to areas of the brain targeted by tox-176-mediated Drd1a-expressing cell death. The robust regional increase in the number of newly synthesized microglia is likely to reflect an inflammatory response elicited by the primary death of Drd1a-expressing cells. Microglia, with their elaborate processes, respond to the presence of necrotic tissue by phagocytosing and removing debris [10-13]. Microglia have other roles including modulation of synaptogenesis and neurogenesis [14], processes that may be particularly relevant in our models of neurodegeneration.

There was a marked enhancement of the number of dividing astrocytes in the cortex and periventricular region of young mutant mice belonging to the GFP-global line although, no such enhancement was observed in mutant mice belonging to the striatal line. Astrocytes are known to participate in long-term brain repair and maintenance [15-18]. Their functions include the release of trophic factors that influence neurite growth, and the formation of a glial scar through the process of reactive gliosis. The survival of neurons is known to be dependent on astrocyte functions such as free radical scavenging and glutamate uptake/release.

In order to determine why a pleiotropic proliferative/repair response appears to have been activated in mutant mice from the GFP-global line but not from striatal line mutant mice, it is worthwhile comparing the time of onset of death of Drd1a-expressing cells in each line. The GFP-global line is known to undergo death of Drd1a-expressing cells at an earlier stage than the striatal line. The death of these cells commences at about 1–2 weeks of age in the GFP-global line, following Cre activation [3,6], and 5 weeks of age in the striatal line [4]. Our GFP-global mutant and GFP-control mice, therefore, had been subjected to several weeks of cell death prior to the commencement of the BrdU injection regime at 4 weeks of age. In contrast, the death of Drd1a-expressing cells would not have commenced in striatal line mutant mice until 5 weeks of age, which is at a point halfway through the 2-week BrdU injection regime administered to these animals commencing at 4 weeks of age. The death of these cells would, therefore, have been relatively advanced in GFP-global as compared with striatal mutant mice. It is conceivable that the enhanced number of dividing astrocytes in the mutant mice belonging to the GFP-global line reflected the comparatively early time of death of the Drd1a-expressing cells in this line, which allowed a greater time for a proliferative astrocyte response to develop.

The presence of BrdU+/NeuN+ double positive cells in the cortex, striatum and periventricular regions of young control and mutant mice belonging to the GFP-global and striatal lines confirmed that neurons were generated from dividing cells in the post-natal period in all lines. The number of BrdU+ neurons was increased relative to control mice in the striatum and periventricular region of GFP-global mutant mice but not in striatal line mutant mice. These results are consistent with published reports indicating that while constitutive neurogenesis occurs throughout the post-natal brain [19], albeit at low levels [20-22], enhanced neurogenesis in response to the loss of Drd1a-expressing cells in our study was restricted to the GFP-global line. This result would appear to correlate with a comparatively subtle orofacial phenotype observed in global line mutant as compared with striatal line mutant mice [23]. A caveat for the observed difference between the GFP-global and striatal lines may be that the temporal profile of Cre-expression and therefore tox-176 mediated death of Drd1a-expressing cells in the striatal line was incompatible with a fully evolved proliferative response to become manifest within the time frame of the study design.

The generated NeuN+ cells are likely to do one of two things. First, they may differentiate into Drd1a-expressing striatal medium spiny GABAergic projection neurons or Drd1a-expressing cortical glutamatergic corticostriatal projection thereby replacing cells that are killed by tox-176 expression. Owing to their molecular signature, these cells are likely to ultimately suffer the same fate as the cells they replace. Second, the generated cells may differentiate into Drd2-expressing striatal projection neurons, non-Drd1a-expressing striatal interneurons or cortical non-Drd1a-expressing pyramidal cells. Expansion of the non-Drd1a-expressing compartments would thereby functionally compensate for the primary loss of Drd1a-expressing cells. We have evidence for up-regulated D2-compartment expression for the global-ablation mutant mice [3,24,25] lending support to this compensation hypothesis. An experiment quantifying BrdU+ cells co-expressing Drd2 in young BrdU injected global-mutant mice on a D2-GFP reporter background would directly address this question.

The number of dividing Olig2+ cells was increased in the cortex and striatum of young mutant mice from the GFP-global line but not young mutant mice from the striatal line. Olig2 is a transcription factor found in cells that differentiate into oligodendrocytes [26]. The differentiated cells supply the axons of neurons with their myelin coats, an important function that correlates with neurogenesis and neural maturation/remodelling as newly generated axons require myelin [27]. It is conceivable that our finding of enhanced numbers of dividing Olig2+ cells in the mutants from the GFP-global line reflects the necessary partnership between the generation of new oligodendrocytes and the generation of new neurons, neuronal processes and neuronal interconnections in these animals.

The expression of Olig2 is not confined to the differentiation path that produces mature oligodendrocytes. Olig2+ cells may also differentiate into neurons [28,29], ependymal cells [30] and astrocytes [31,32]. It is conceivable that dividing Olig2+ cells in the cortex and striatum of the mutants from the GFP-global line may ultimately differentiate into neurons or astrocytes in these regions of the brain, and thus contribute to the observed up-regulation of these types of cells in response to tox-176-mediated death of Drd1a-expressing cells.

In the cortex of control and mutant mice for the GFP-global and striatal lines, virtually 100% of the dividing cells were identified on the basis of marker expression, as microglia, astrocytes, neurons or oligodendrocytes. However, in the striatum and to a greater extent in the periventricular region, a large percentage of the dividing cells were negative for all markers suggesting that an increasing proportion of cells were progenitor cells that remained uncommitted to any particular lineage. Judging from the distribution profile of identifiable dividing cells in our study, it would appear that new cells that were manufactured in the sub-ventricular zone of the periventricular region divided and differentiated as they migrated through the striatum and to the cortex. By the time they reached the cortex, differentiation was more or less complete, and thus based on co-expression of lineage specific markers, all BrdU+ cells were readily assigned a phenotype. We propose that some of the phenotype negative BrdU+ cells in the striatum and periventricular region may consist of immature neurons that are yet to express NeuN. Validation of this idea would require further experiments such as; a time course analysis of the regional distribution of BrdU positive cells after BrdU-pulsing, staining for co-expression of BrdU-positive cells with markers seen in migrating and differentiating neuronal precursors such as doublecortin and PSA-NCAM [33], or an analysis of Ki67-positive cell distribution in a younger mutant cohort. Neurogenesis occurs in the adult mammalian brain, including that of humans [34,35]. Neurogenesis is known to occur in the sub-ventricular zone [36,37], the region of the brain that lines the lateral ventricular cavity. It is also possible that rather than originating from periventricular zone stem cells, NeuN/BrdU double positive cells could originate in situ from quiescent stem cells found in the striatum.

Young global and striatal ablation mutants assessed using BrdU as a proliferative marker displayed a marked up-regulation in the number of dividing cells. In contrast, older mice of the global and cortical mutant lines showed markedly reduced Ki-67+ cell numbers when compared to control mice. Experimental confirmation of this finding would involve a direct comparison of the proliferative profile in 4 week and 80 weeks old mice using established Ki-67 immunofluorescence and BrdU protocols. Our finding of reduced Ki-67+ cell numbers in older mutant lines appears to be at odds with some published studies, which underscore an increased proliferative response in humans with HD and rodent HD models [38,39], although other studies in HD models have identified region specific impaired neurogenesis. Decreased hippocampal dentate gyrus neurogenesis was seen in the transgenic YAC128 model [40]. Olfactory bulb neurogenesis was down-regulated in the hippocampus [41] and olfactory bulb [42] of R6/2 transgenic HD mice and striatal cell neurogenesis was impaired in a HD knock-in line [43]. However, it is important to note the age (i.e. 80 weeks) of the mice used in this study. The comparative difference between mutant and control mice may relate to the advanced age of the mice used in this study. We postulate that the aged mutant brain is unable to mount an effective proliferative response [44] owing to paradigm driven increased proliferative demand in early adulthood and ultimate stem cell pool depletion. There is ample evidence for age-related stem cell depletion in the CNS [19,45]. Although neural stem cells isolated from the subventricular zone of aged animals are able to divide and differentiate into functional neurons and other neural lineages, they do so with reduced efficiency [45]. A large number of factors may impact on this age-related decline in functional stem cell depletion such as changes in the neurogenic niche and neural progenitor cell specific variables such as altered gene transcription and telomerase activity [19].

Another explanation for the reduced number of Ki-67+ cells in the mutant mice is the possibility that the proliferative pool contains Drd1a-expressing cells. In this scenario, neural precursor cells that express Drd1a in a transient fashion undergo tox-176-mediated death before they have an opportunity to enter a permissive phase of the cycle and express Ki-67. Cre expression would occur only if the CamKIIa or EMX1 transgenes are expressed in neural precursor cells of the global and cortical lines respectively. If Drd1a is transiently expressed in these cells, then Cre must be expressed before Drd1a to enable Cre-mediated recombination of LoxP sites and Drd1a promoter-driven production of tox-176. If neural progenitor cells express Drd1a, death of these cells will ensue resulting in the absence of a proliferative response in this paradigm and the uniformly low Ki-67+ cell numbers seen in the mutant mice. There are two strong counter arguments. The first is that we did not identify any GFP/Ki-67 double positive cells in GFP-control mice and the second is that the BrdU data from young mice show a clear increase in proliferative response in mutant mice that are designed to shed Drd1a-expressing cells at the time of Drd1a receptor expression.

A further possibility is that the difference in Ki-67+ cell numbers between mutant and control mice in the rostral striatum is not an indication of a decrease in proliferation in the mutant mice, but, rather reflects an increase in migration and terminal differentiation of proliferating cells in response to the molecular pathology in mutant mice. It may be the case that there is, indeed, an increase in proliferation in mutant mice but that the proliferating cells are also differentiating and migrating from the periventricular region/medial striatum towards areas of high cell death density. If this were the case, and considering that Ki-67 is only a marker for early cell division [46], these differentiating and migrating cells would not be labelled with Ki-67. This is an unlikely explanation given the BrdU data indicate a gradient of phenotype-negative cells maximal in the periventricular region.

## Conclusion

We used exogenous (BrdU) and endogenous (Ki-67) markers of cell proliferation to determine the profile (distribution and quantity) of dividing cells in young (4-8-weeks of age) and old (80-weeks of age) mouse models of basal ganglia diseases. In terms of the young mice, we found that mutants from the GFP-global line have a greater number of newly generated cells than control mice in the relevant brain regions. Mutant mice from the striatal line have a greater number of dividing microglia than control mice but there is no increase in the number of newly generated neurons, oligodendrocytes or astrocytes. We have, therefore, demonstrated that genetically programmed non-traumatic and non-infectious targeted deletion of Drd1a-expressing cells in the post-natal brain provokes a proliferative response that involves a large number of cell lineages. Paradoxically, the number of dividing cells lining the lateral ventricles-as defined by Ki-69 expression-is reduced in older mutant mice belonging to global and cortical lines. We postulate that this may relate to stem cell pool depletion.

## Methods

### Mouse lines

All mice were housed 2–5 per cage with food and water available ad libitum in a temperature-controlled environment with a 12 hours (h) light/dark cycle. The Howard Florey Institute animal ethics committee approved all experiments.

#### GFP-global line

CD1-backcrossed heterozygous mice containing the silenced tox-176 gene [5] were mated with Drd1a/GFP mice from the GENSAT BAC Transgenic Project [47]. Offspring carrying both the Drd1a-GFP transgene and the silenced tox-176 gene were then mated with a heterozygous C57BL/6JArc-backcrossed Cam Kinase IIa promoter driven Cre transgenic line [6]. GFP-global mutant mice were defined as triple transgenic (Drd1a-GFP/Cam Kinase IIa/Tox) whereas GFP-control mice were littermates that were transgenic for the Drd1a-GFP gene alone.

#### Global line

CD1-backcrossed heterozygous mice containing the silenced tox-176 gene [5] were mated with a heterozygous C57BL/6JArc-backcrossed Cam Kinase IIa promoter driven Cre transgenic line [6]. Mutant mice were heterozygous for both transgenes. Littermates that were not transgenic for either transgene were defined as control mice.

#### Cortical line

CD1-backcrossed heterozygous mice containing the silenced tox-176 gene [5] were mated with a heterozygous C57BL/6JArc-backcrossed EMX1 promoter driven Cre transgenic line [48]. Cortical line mutant mice were heterozygous for both transgenes. Wild type mice from the Drd1a/Tox × Cam Kinase IIa/Cre mouse cohort were used as littermate controls.

#### Striatal line

CD1-backcrossed heterozygous mice containing the silenced tox-176 gene [5] were mated with a heterozygous C57BL/6JArc-backcrossed DARPP-32 promoter-driven Cre transgenic line [4]. Mutant mice were heterozygous for both transgenes. Control mice were littermates that were not transgenic for either transgene.

### BrdU injections

Four-week old female mice of the striatal (*n*=4 mutants, *n*=4 controls) and GFP-containing global (*n*=4 mutants, *n*=4 controls) lines were injected intraperitoneally with BrdU (50 mg/kg). Each animal was injected twice daily, 8 h apart, for 2 weeks. Following the last injection, animals were returned to their cages and a further period of two weeks allowed to elapse before the animals were killed with an injection of 0.2 mls of a 1:4 dilution of Lethobarb (Virbac, Australia Limited) in normal saline and then perfused transcardially with 0.1M phosphate buffered saline (PBS) containing heparin (1,000 U/kg; 37°C) followed by an ice-cold mixture consisting of 4% paraformaldehyde containing 7% picric acid diluted in 0.1M PBS (pH 7.4). and the brains removed for BrdU analysis using immunohistochemistry. A number of published BrdU injection protocols are available [49][50][51]. Single injections are used if the aim is to precisely date cell birth. This was not a requirement of our study. Indeed, we chose a protocol involving multiple BrdU injections, as we wanted to ensure adequate labeling of many cells undergoing cell division over an extended period of time. We were concerned firstly, about the inherently low rate of cell turnover in tissues such as the striatum and cortex, and secondly that transient expression of Drd1a in progenitor cells may compromise cell viability and further reduce the number of BrdU+ cells given the evidence for a role of D1-like receptors activation on the division of striatal progenitor cells [52].

### Ki-67 analysis

Eighty-week old mice of the global (*n*=4 mutants, *n*=4 controls), GFP-global (*n*=4 mutants, *n*=4 controls) and cortical (*n*=4 mutants, *n*=4 controls) lines were killed with an injection of 0.2 mls of a 1:4 dilution of Lethobarb (Virbac, Australia Limited) in normal saline and then perfused transcardially with 0.1M PBS containing heparin (1,000 U/kg; 37°C) followed by an ice-cold mixture consisting of 4% paraformaldehyde containing 7% picric acid diluted in 0.1M PBS (pH 7.4). and the brains removed for Ki-67 analysis using immunohistochemistry.

### Tissue processing

Following tissue fixation, brains were removed and immersed in the same fixative solution for 90 min before being transferred to 10% sucrose and stored for a further 96 h at 4°C. Brains were subsequently frozen using CO_2_ (BOC Gases).

Frozen brains were sectioned coronally (14 μm) using a cryostat (Leica CM 1850). Consecutive sections between bregma 1.18 mm and −0.46 mm were collected onto chrome alum-gelatin-coated glass slides, air dried, and stored at −20°C. The distances from bregma [53], which spanned the striatum, were determined using the corpus callosum, anterior commissure and hippocampus as reference points. In the case of 80-week-old mice, sections were selected from 7 levels: 1.18, 0.50, 0.26, 0.14, -0.10, -0.22 and −0.46 mm from bregma. In the case of 4-week-old mice, sections were selected from 5 levels: 1.18, 0.62, 0.26, 0.02 and −0.46 mm from bregma.

### Immunohistochemistry

Primary antibodies included rabbit anti-Ki-67 (1/200; Thermo Scientific, USA) for dividing cells expressing Ki-67, rat anti-BrdU (1/200; Novus Biologicals, USA) for dividing cells incorporating BrdU, chicken anti-GFP (1/1000; Sapphire Bioscience, Australia) for GFP-containing neurons, rabbit anti-Olig2 (at a dilution of 1/200) for oligodendrocytes, rabbit anti-Iba1 (1/200; Wako, Japan) for microglia and macrophages, rabbit anti-S100β (1/1,500; Abcam, USA) for astrocytes, and mouse anti-NeuN (1/500; Millipore, USA) for neurons. Secondary antibodies included goat anti-rabbit IgG (1/200; Alexa Fluor 594, Invitrogen, USA), goat anti-rat IgG (1/200; Alexa Fluor 488, Invitrogen, USA), goat anti-chicken IgG (1/200; Alexa Fluor 488, Invitrogen, USA) and biotinylated horse anti-mouse IgG (1/200; Vector) linked to streptavidin-549 (1/200; Jackson).

#### Double labelling using rat anti-BrdU and rabbit-derived antibodies

Sections were double-labelled over a two-day period using a rat anti-BrdU and a selected rabbit-derived antibody against Olig2, Iba1 or S100β. On the first day, sections on slides were placed in 0.1M PBS for 10 min and then immersed in a solution containing a 1:1 volume ratio of 0.1M PBS and formamide. Following incubation at 65°C for 2 h, sections were rinsed three times for 5 min in O.1M PBS. A solution of 2 M hydrochloric acid was added and incubated at 37°C for 30 min and then sections rinsed in 0.1M PBS. A solution of 0.1 M sodium borate was then added to each section and incubated at room temperature for 15 min then rinsed in 0.1M PBS.

Primary antibodies were diluted in a solution consisting of 0.1M PBS containing 10% goat serum and 0.4% triton-X100. The primary antibody mix was added to each section and the sections placed in a humidified box, incubated overnight at room temperature and then rinsed in 0.1M PBS. 0.1M PBS containing 10% goat serum and 0.4% triton-X100 was then added to each section for 15 min at room temperature. Sections were then covered with goat anti-rat IgG and goat anti-rabbit IgG secondary antibodies diluted in 0.1M PBS containing 10% goat serum and 0.4% triton-X100 and incubated for 3 h at room temperature. Sections were then rinsed in 0.1M PBS (3 × 10 min), cover slipped with fluorescent mounting medium and allowed to dry.

#### Double labelling using rat anti-BrdU and mouse anti-NeuN

The staining protocol required the following modification in the case of double labelling using rat anti-BrdU and mouse anti-NeuN. 0.1M PBS containing 10% goat serum, 10% horse serum and 0.4% triton-X100 was used to dilute all primary and secondary antibodies and streptavidin-549. The secondary antibody for mouse anti-NeuN consisted of a biotinylated horse anti-mouse IgG. The sections were incubated in a mixture of secondary antibodies for BrdU and NeuN for 1 h, rinsed in 0.1M PBS, and then incubated for a further 1 h in streptavidin-549 to amplify the NeuN signal.

#### Double labelling using chicken anti-GFP and rabbit anti-Ki-67

Sections were double-labelled using the primary antibodies chicken anti-GFP and rabbit anti-Ki-67 over a two-day period. Sections on the slides were circled with a wax pen and rinsed three times in 0.1 M PBS for 10 min each time. 0.1 M PBS containing 10% goat serum and 0.3% triton-X100 was then added to each section and incubated for 30 min at room temperature. Following another brief wash in 0.1 M PBS, sections were incubated overnight at 4°C with a mixture of rabbit anti-Ki-67 and chicken anti-GFP antibodies diluted in 0.1M PBS containing 5% goat serum and 0.25% triton X100. Sections were then rinsed (3 x 10 min in 0.1M PBS) and incubated for 3 h at room temperature with a mixture of the secondary antibodies goat anti rabbit IgG and goat anti-chicken IgG diluted in 0.1 M PBS containing 5% goat serum and 0.25% triton X100. Sections were then rinsed in 0.1M PBS (3 × 10 min), coverslipped with fluorescent mounting medium and allowed to dry.

### Fluorescence microscopy

An epifluorescence microscope (Leica DMLB2) and a confocal microscope (Olympus FV 1000) were used to visualize the results of immunohistochemical staining. In the case of epifluorescence microscopy, a HC software package was used to capture images of the right hemisphere of the brain at an objective lens magnification of X20. Images captured under blue excitation (for green emission) and green excitation (for red emission) wavelengths were merged to indicate the presence of double-labelled (yellow) cells. In the case of confocal microscopy, an FV software package was used to capture images at X20 and X60 objective lens magnifications, and Z stacks were used to confirm double labelling.

### Image analysis and cell counting

Images captured with the epifluorescence microscope, and the associated merged images, were opened as TIFF files using the Image Pro Plus 3DS software package. Using the Media Cybernetics program, single-labelled (green or red) and double-labelled (yellow) cells were manually tagged and counted at various levels between 1.18 mm and −0.46 mm from the bregma point, which spans much of the striatum, with the observer blinded to genotype. We quantified cell numbers in the M1 region of the cortex, the dorsal striatum and the periventricular region [53]. The cell numbers were calculated for each of the five pre-determined coronal bregma levels and all counts were performed at the same magnification. Specifically, we quantified cells in the dorsal striatum, which is located ventral to the corpus callosum, with a counting frame measuring 492μm in width and 375μm in height. The counting frame extended ventrally from the junction of the corpus callosum and the striatum. For analysis of the periventricular region, the height of the counting frame was 375μm and the width of the counting frame extended across a distance of 300μm into the striatum from the junction of the striatum and the lateral ventricular wall (in the case of Ki-67+ cells) and across a distance of 300μm taken 150μm on either side of the midpoint of the lateral ventricle (for quantification of periventricular BrdU+ cells). This approach captured BrdU+ cells on either side of the ventricle and within the highly proliferative zone of the septal region. For analysis of the periventricular region, the counting frame was placed immediately ventral of a cell dense dorsomedial region of the striatum that is generally devoid of fibre bundles. We know that this part of the striatum is relatively spared in the striatal model (see Additional file 1: Figure S1). These data are presented either as counts at specific bregma levels for different regions (cortex, striatum or periventricular region) (Figures 3 and 6) or as cumulative counts in which counts at multiple bregma levels are summed for a given region (Figure 2).

As GFP expressing reporter mice were used in this study, it is important to point out, for a number of reasons, that BrdU+ cell counts (determined using green fluorescence) obtained in mice carrying the Drd1a-EGFP BAC transgene reflect the number of cells expressing BrdU and not the number of GFP-expressing cells. First, in our hands, green fluorescence related directly to expression of the Drd1a-EGFP BAC transgene is not apparent without the use of an anti-GFP antibody. Second, the cellular pattern of Drd1a-GFP and BrdU expression is different (compare panels A-L in Figure 1 with panels B-D and F-H in Figures 8). GFP expression (when detected using anti-GFP immunofluorescence) involves the neuropil and demarcates the entire neuronal cell, as the GFP protein is present throughout the cytoplasm whereas BrdU is exclusively nuclear. Third, reporter mice (i.e. Drd1a-GFP BAC transgene expressing littermates) were used as controls in this experiment. If we were counting GFP expressing cells then there would be higher cell counts throughout the brain (identical to what is seen in Figure 8) that would almost certainly swamp any genotype specific BrdU signal, indeed, the number would be greater in GFP-control mice as they have a greater number of GFP expressing cells. Fourth, we are unable to identify GFP expression in the cortex of Drd1a-EGFP BAC transgene expressing reporter mice despite deploying anti-GFP antibody (presumably because of the low Drd1a-GFP level of expression in this subpopulation) whereas BrdU-expressing cells are readily apparent (see Figures 1A, D, E, H).

### Statistics

Statistical analysis of the numbers of single-labelled and double-labelled cells was performed using the GraphPad^R^ Prism software program, version 5.00 (GraphPad^R^ Software Inc., San Diego, CA, USA). Data were expressed as mean ± standard error of the mean (SEM). A two-way analysis of variance (ANOVA) was used to assess comparisons, the two variables being genotype and bregma level. A Bonferroni post-hoc test was used if appropriate. Comparisons were deemed significant at *P* < 0.05.

## Abbreviations

HD: Huntington disease; Drd1a: D1 dopamine receptor; CamKIIa: Calmodulin kinase IIa; DARPP-32: Dopamine and adenosine 3’, 5’-cyclic monophosphate-regulated phosphoprotein, 32kDa; BrdU: 5’-bromo-2’-deoxyuridine; GFP: Green fluorescent protein; ANOVA: Analysis of variance; PBS: Phosphate buffered saline.

## Competing interests

The authors declare no competing interests.

## Authors’ contributions

AS performed BrdU studies including immunohistochemical phenotyping and generated the first draft of the manuscript. KR performed Ki67 study and contributed to preparation of the manuscript. AHK generated global line colony and contributed to Additional data. JM contributed experimentally to the generation the Drd1a-tox-176 Floxed line and performed BrdU and Ki67 studies. COL performed some immunohistochemical cell phenotyping of BrdU studies. ME generated the DARPP-32 mouse used in the production of the striatal line. GS generated the CamKIIa/Cre mouse used in the production of the global lines. AJL was involved in study design and contributed to preparation of the manuscript. JD generated the Drd1a-tox-176 Floxed line, designed the study and drafted the manuscript. All authors read and approved the final manuscript.

## Supplementary Material

Additional file 1: Figure S1 Fluorescence microscopy highlighting densely packed Drd1a-GFP-positive cells in the dorsomedial striatum. Photomicrograph of striatal line Drd1a-GFP WT control mouse brain (GFP cells labeled green) (**A**) and striatal line mutant mice on a GFP genetic background (**B**). Drd1a-GFP-positive cells are abundantly expressed throughout the striatum in the GFP-control mouse brain and significantly lost in GFP-striatal mouse brain. Dotted line outlines the dorsomedial striatum that remains relatively densely packed with Drd1a-GFP-positive cells in both lines. Scale bar = 100 μm. Click here for file
