# Supplementary material for: Phenotyping dividing cells in mouse models of neurodegenerative basal ganglia diseases
Source: BMC Neurosci. 2013 Oct 3;14:111. doi: 10.1186/1471-2202-14-111 (PMC3851877; doi:10.1186/1471-2202-14-111)
Supplement: Additional file 1: Figure S1 — Fluorescence microscopy highlighting densely packed Drd1a-GFP-positive cells in the dorsomedial striatum. Photomicrograph of striatal line Drd1a-GFP WT control mouse brain (GFP cells labeled green) (A) and striatal line mutant mice on a GFP genetic background (B). Drd1a-GFP-positive cells are abundantly expressed throughout the striatum in the GFP-control mouse brain and significantly lost in GFP-striatal mouse brain. Dotted line outlines the dorsomedial striatum that remains relatively densely packed with Drd1a-GFP-positive cells in both lines. Scale bar = 100 μm. [file 1471-2202-14-111-S1.pdf]

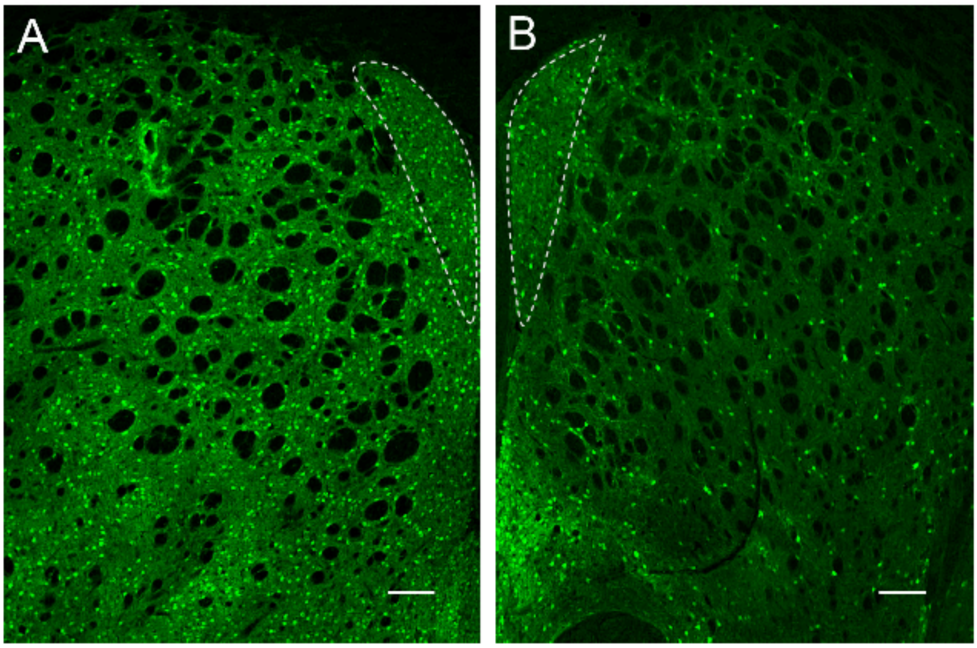

Photomicrograph of striatal line Drd1a-GFP WT control mouse brain (GFP cells labeled green) (A) and striatal line mutant mice on a GFP genetic background (B). Drd1a-GFP-positive cells are abundantly expressed throughout the striatum in the GFP-control mouse brain and significantly lost in GFP-striatal mouse brain. Dotted line outlines the dorsomedial striatum that remains relatively densely packed with Drd1a-GFP-positive cells in both lines. Scale bar = 100μm.
